# Factors affecting seizure recurrence in the emergency department

**DOI:** 10.1016/j.heliyon.2024.e26833

**Published:** 2024-02-22

**Authors:** Umit Can Dolek, Mustafa Gokce, Mehmet Muzaffer Islam, Serdar Ozdemir, Gokhan Aksel, Abdullah Algin

**Affiliations:** a Khoja Akhmet Yassawi International Kazakh-Turkish University, Medical School, Emergency Department, Turkistan, Kazakhstan; b Emergency Medicine Department, University of Health Sciences, Umraniye Training and Research Hospital, Istanbul, Turkiye; c Khoja Akhmet Yassawi International Kazakh-Turkish University, Medical School, Department of Neurology, Turkistan, Kazakhstan

**Keywords:** Recurrent seizure, Epilepsy cluster, Immediate seizure, Recurrent epilepsy

## Abstract

**Background:**

Information on Emergency Department (ED) follow-up of patients presenting with epileptic seizures is limited.

**Objectives:**

It was planned to investigate the factors affecting the recurrence of epileptic seizures in the follow-up of patients presenting to the ED with the complaint of epileptic seizures.

**Materials and methods:**

This prospective, observational, single-center study was carried out in an adult population presenting to the ED. The study included patients older than 18 years of age presenting to the ED with the complaint of epileptic seizures.

**Results:**

Of the 205 patients included in the study, 68 (33.2%) had seizure recurrence during the 6 h. In the univariable analysis, advanced age, prolonged post-ictal duration, increased seizure duration, generalized tonic clonic seizure, alcohol consumption within past 24 h, hypertension, coronary artery disease, Alzheimer's disease, prior ischemic cerebrovascular disease, low Glascow Coma Scale (GCS), high glucose, high C-Reactive Protein, high phosphorus, low potassium, high blood urea nitrogen, high lactate, increased anion gap, high osmolarity were statistically significant in predicting recurrent seizure recurrence within 6 h. According to the logistic regression, postictal duration, GCS score, and age were independent predictors in our model. The cut-off value of postictal duration in predicting seizure recurrence at the highest sensitivity (66.2%) and specificity (89.8%) was 22.5 min.

**Conclusion:**

A prolonged postictal state, low GCS score, advanced age may be an indication of seizure recurrence. Therefore, patients with a long postictal duration, low GCS score, advanced age should be followed up more carefully in terms of recurrent seizures in the ED.

## 1 Introduction

Epilepsy, one of the most common neurological disorders, affects approximately 70 million people worldwide [1]. Patients with epilepsy often experience recurrent seizures or seizure clusters despite treatment with antiepileptic drugs [2,3]. There is no consensus on the definition of seizure clusters, and they are not included in the International League Against Epilepsy classification [4]. While some studies define seizure clusters as two or more seizures occurring within four or 6 h, others indicate that three or more seizures that occur within 24 h can be classified as seizure clusters [5,6]. There is also insufficient evidence for clinicians to predict which patients may develop seizure clusters. Although most clinicians consider that a follow-up period of four to 6 h after seizures may be sufficient for the discharge of patients with epilepsy, there is still no common scientific consensus on this issue [7,8]. Various studies have attempted to define factors affecting recurrence in patients with epileptic seizures in the early period. In a study carried out with 956 patients, Lossius et al. showed that the use of dual antiepileptic drugs and an age over 50 years old were the factors affecting epileptic seizure recurrence [9]. Similarly, Choquet et al. found that abnormal neurological examination findings at the time of admission, a low Glasgow Coma Scale (GCS) score, a high plasma glucose level, and alcoholism might be risk factors for epileptic seizure recurrence [10]. However, results seem to vary depending on differences in study designs. In our study, we aimed to reveal the possible risk factors for epileptic recurrence in patients presenting to the emergency department with epileptic seizures.

## 2 Materials and Methods

### 2.1 Study population and setting

This prospective, observational, and descriptive study was carried out between October 1, 2020, and September 30, 2021, at the University of Health Sciences, Umraniye Training and Research Hospital, which has approximately 350,000–500,000 adult patient admissions to the emergency department annually.

Patients older than 18 years of age presenting to the emergency department with the complaint of epileptic seizures with or without a new onset were included. Patients with incomplete data, those that refused to participate in the study, those that had had their last seizure more than 1 h before, and those that refused treatment and examinations during the 6-h observation period were excluded from the study. Seizure treatment was performed according to the routine clinical approach of the hospital, i.e., the patients’ treatment was not altered during the study. We only observed the patients for 6 h and explored etiological factors that could cause seizure recurrence.

### 2.2 Definition of terms

The diagnosis of epileptic seizures was made using the patients' detailed history and clinical examination findings. The end of postictal duration was determined based on patients’ recovery of consciousness, improved orientation, and correct and appropriate responses to commands.

### 2.3 Data collection

For each patient included in the study, a three-part data form was completed by an emergency medicine specialist or resident providing care for the patient. In the first part of the data form, seizure type, seizure duration, alcohol or substance use within the last 24 h, expression of emotional stress, and comorbid diseases, such as hypertension, diabetes mellitus, coronary artery disease, and chronic obstructive pulmonary disease were questioned. In the second part of the form, postictal duration, fever, pulse, systolic-diastolic blood pressure, oxygen saturation, GCS score, and presence of major neurological deficits determined by the doctor were recorded. The third part included laboratory findings obtained at the time of the patients' arrival, including hemoglobin, glucose, C-reactive protein, calcium, magnesium, phosphorus, sodium, chlorine, potassium, creatine kinase, albumin, pH, carbon dioxide, lactate, base deficit, anion gap, blood urea nitrogen, and osmolarity values. The patients were observed in the emergency department for at least 6 h, and those with and without seizure recurrence were compared. Patients were not routinely administered benzodiazepines and Antiepileptic Drugs, either en route to the ED or during their stay, but this data was not available for this study. The clinic where the study was conducted has a 12-bed intensive care unit (referred to as the red zone in some emergency departments.). The emergency intensive care unit has one attending physician, one senior resident, two junior residents and six nurses on duty. The consciousness of patients experiencing seizures was regularly monitored until the postictal periods ended, and during this process, it was documented by the doctors or nurses present in the area. The GCS scores of the patients were measured at the time of their emergency service admission. The GCS value at admission was evaluated as a predictive factor for recurrent seizures.

## 3 Statistical analysis

The Statistical Package for the Social Sciences (SPSS) for Windows, v. 26.0 software was used for the statistical analysis of the research data. Categorical data were expressed as frequency and percentages. Since all the continuous data were inconsistent with a normal distribution, they were expressed as median (interquartile range of 25–75%). The chi-square test was used to compare categorical data, and Fisher's exact test was employed where necessary. The Mann-Whitney *U* test was used for the intergroup comparison of continuous data. Among the variables that were found to be statistically significant in the univariable analysis, those that were considered to have a logical relationship with outcome in light of the literature were included in the regression model. No variable was excluded from the model because there was no high correlation between the variables in the correlation matrix. The fit of the model was tested with the Hosmer-Lemeshow test. The receiver operating characteristic (ROC) test was used to calculate the diagnostic value of the variables in predicting seizure recurrence, and their area under the curve values were calculated. After determining the cut-off value providing the highest sensitivity and specificity using Youden's index, the positive predictive value, negative predictive value, positive likelihood ratio, negative likelihood ratio, and accuracy were calculated at the optimal cut-off values. Statistical significance was accepted as p < 0.05 in all results.

## 4 Ethical considerations

Approval was obtained from the ethics committee of University of Health Sciences, Umraniye Training and Research Hospital according to local regulations (number: B.10.1TKH.4.34. HGP.0.01/314). All participants provided written informed consent for participation in the study.

## 5 Results

**Fig. 1:**
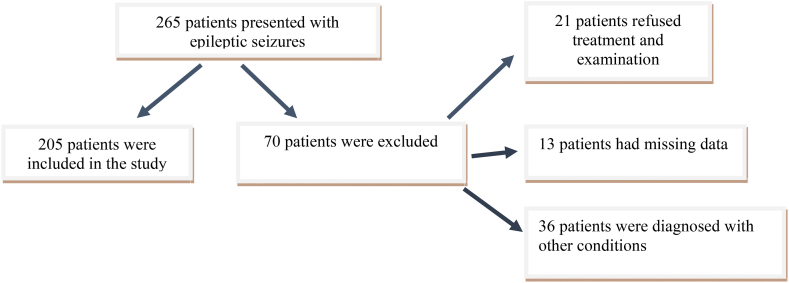
Patient flow chart.

After excluding 13 patients with missing data, 36 patients with other diagnoses, such as pseudo-seizure, syncope, and migraine, and 21 patients that refused treatment or examinations during their follow-up, the data of a total of 205 patients were included in the statistical analysis (Fig. 1). When the entire study sample was examined in terms of basic descriptive characteristics, it was observed that the median age of the patients was 40 (27–58) years, and 121 (59%) were male. Sixty-eight (33.2%) patients had seizure recurrence in the emergency department within 6 h of observation. The number of hospitalized patients was 48 (23.4%), and 14 (6.8%) of these patients were admitted to the intensive care unit. The median postictal period was 10 (5–30) minutes. The main descriptive characteristics of the patients and the results of the univariable analysis are summarized in Table 1.

**Table 1:** Basic descriptive data of the all patients and results of the univariable analysis.

	n (%)/median (25–75%) interquartile range)	Patients without seizure recurrence	Patients with seizure recurrence within 6 h	P value
**Median Age**	40 (27–58)	34 (24–48)	56 (41–72)	**<0.001**
**Gender (Male)**	121 (59%)	84 (61.3)	37 (54.4)	0.344
**Seizure duration (sec)**	60 (20–300)	60 (15–180)	135 (38–300)	**0.002**
**Seizure Type (GTC)**	183 (89.2%)	127 (92.7)	55 (80.9)	**0.012**
**Postictal duration (min)**	10 (5–30)	10 (5–10)	48 (15–120)	**<0.001**
**Alcohol consumption within past 24 h**	5 (2.4)	1 (0.7)	4 (5.9)	**0.042**
**Autism/MR/CP**	12 (5.9)	6 (4.4)	6 (8.8)	0.168
**Central nervous system Tumor**	17 (8.3)	8 (47.1)	9 (52.9)	0.071
**Febrile convulsion history**	5 (2.4)	3 (2.2)	2 (2.9)	0.537
**Diabetes mellitus**	20 (9.8)	10 (7.3)	10 (14.7)	0.092
**Hypertension**	26 (12.7)	11 (8)	15 (22.1)	**0.004**
**Coronary artery disease**	21 (10.2)	7 (5.1)	14 (20.6)	**0.001**
**COPD**	9 (4.4)	6 (4.4)	3 (4.4)	0.992
**Malignancy**	11 (5.4)	6 (4.4)	5 (7.4)	0.281
**Prior ischemic CVD**	12 (5.9)	4 (2.9)	8 (11.8)	**0.015**
**Alzheimer's disease**	11 (5.4)	3 (2.2)	8 (11.8)	**0.007**
**Other comorbidities**	7 (3.4)	5 (3.6)	2 (2.9)	0.574
**GCS score**	15 (15–15)	15 (15–15)	13 (11–15)	**<0.001**
**Systolic blood pressure (mmHg)**	123 (112–140)	121 (111–135)	128 (115–147)	0.077
**Diastolic blood Pressure (mmHg)**	75 (66–82)	75 (66–82)	76 (67–81)	0.768
**Pulse (Beats/min)**	85 (80–100)	87 (80–101)	84 (80–99)	0.400
**Oxygen saturation**	97 (95–99)	98 (96–99)	97 (94–99)	0.130
**Fever °C**	36.4 (36–36.6)	36.4 (36.2–36.6)	36.2 (36–36.6)	0.259
**Hemoglobin (g/dL)**	13.6 (12.2–15.1)	13.7 (12.4–15.2)	13.2 (12.2–14.6)	0.175
**Glucose (mg/dL)**	106 (92–137)	104 (90–127)	126 (95–149)	**0.002**
**CRP (mg/L)**	1.1 (0.3–4.4)	0.9 (0.3–3.6)	2.2 (0.6–8)	**0.004**
**Calcium (mg/dl)**	9 (8.7–9.3)	9 (8.7–9.3)	9 (8.8–9.4)	0.705
**Magnesium (mg/dl)**	2 (1.8–2.1)	2 (1.9–2.1)	1.9 (1.8–2.1)	0.174
**Phosphorus (mg/dl)**	3.2 (2.6–3.6)	3 (2.6–3.6)	3.4 (2.9–3.8)	**0.027**
**Sodium (mEq/L)**	138 (137–140)	138 (137–139)	139 (136–141)	0.210
**Chlorine (mEq/L)**	103 (100–105)	103 (101–105)	101 (99–105)	0.090
**Potassium (mEq/L)**	4.3 (4–4.5)	4.3 (4.1–4.5)	4.1 (3.8–4.4)	**0.004**
**Creatine kinase** **(U/L)**	106 (70–166)	110 (75–163)	87 (46–199)	0.081
**Albumin (g/L)**	44 (40–46)	44 (40.1–46.1)	43 (39.1–45.6)	0.240
**BUN (mg/dL)**	27 (21–33)	26 (21–30)	30 (21.9–40.1)	**0.021**
pH	7.36 (7.32–7.39)	7.36 (7.33–7.39)	7.35 (7.30–7.41)	0.501
**pCO**_**2**_	42.3 (37–47.7)	42.6 (37.9–47.1)	41.6 (35.5–48)	0.491
**HCO**_**3**_	22.9 (20–24.5)	22.9 (21–24.6)	22.8 (19.5–24.4)	0.114
**Lactate (mmol/L)**	2.9 (1.85–5.3)	2.6 (1.8–4.9)	3.6 (2.1–6.4)	**0.018**
**Base deficit (mEq/L)**	−0.85 (−4.17–0.9)	−0.7 (−3.1 – 0.95)	−1.1 (−5.4 – 0.9)	0.113
**Anion gap (mEq/L)**	−12.9 (-17–10)	−12.4 (−16–−10)	−13.8 (−21.4–−11.4)	**0.023**
**Osmolarity (mOsmol/L)**	295 (289–301)	294 (288–299)	298 (291–305)	**0.008**

Sec: second, GTC: generalized tonic-clonic seizures, min: minute, MR: mental retardation, CP: cerebral palsy, COPD: chronic obstructive pulmonary disease, CVD: cerebrovascular disease, GCS: Glasgow Coma Scale, CRP: C-reactive protein.

A total of eight variables were included in the regression model. The variables in this model did not show a high correlation with each other. The model was found to have a good fit when analyzed with the Hosmer-Lemeshow test (p = 0.116). The tolerance of all the predictors was above 0.1, indicating that there was no multicollinearity. The model was able to explain 61.5% of all the variance (Nagelkerke R^2^ = 0.615) and accurately classified 88.3% of all the patients. Postictal duration, GCS score, and age were found to be the independent predictors in our model (p < 0.001, p < 0.001, and p = 0.010, respectively). The predictor that most contributed to the regression model was postictal duration (Wald = 16.158). The results of the logistic regression are summarized in Table 2.

**Table 2:** Multivariable logistic regression results.

	Beta-coefficients	Wald statistics	OR (95% CI)
**Postictal duration (mins)**	0.023	16.158	**1.024 (1.012–1.035)**
**GCS**	−0.688	12.161	**0.502 (0.341–0.740)**
**Age**	0.035	6.681	**1.035 (1.008 to 1.063)**
**Alcohol consumption within past 24 h**	2.4	3.283	11.019 (0.822–147.7)
**Lactate (mmol/L)**	0.072	1.742	1.075 (0.966–1.196)
**Seizure duration (secs)**	0.001	1.010	1.001 (0.999–1.003)
**Prior ischemic CVD**	0.608	0.542	1.836 (0.364–9.261)
**Glucose (mg/dL)**	0.002	0.189	1.002 (0.993–1.011)
**Constant**	7.358	5.698	–

CVD: cerebrovascular disease, GCS: glasgow coma scale, OR: odds ratio, CI: confidence interval.

**Fig. 2:**
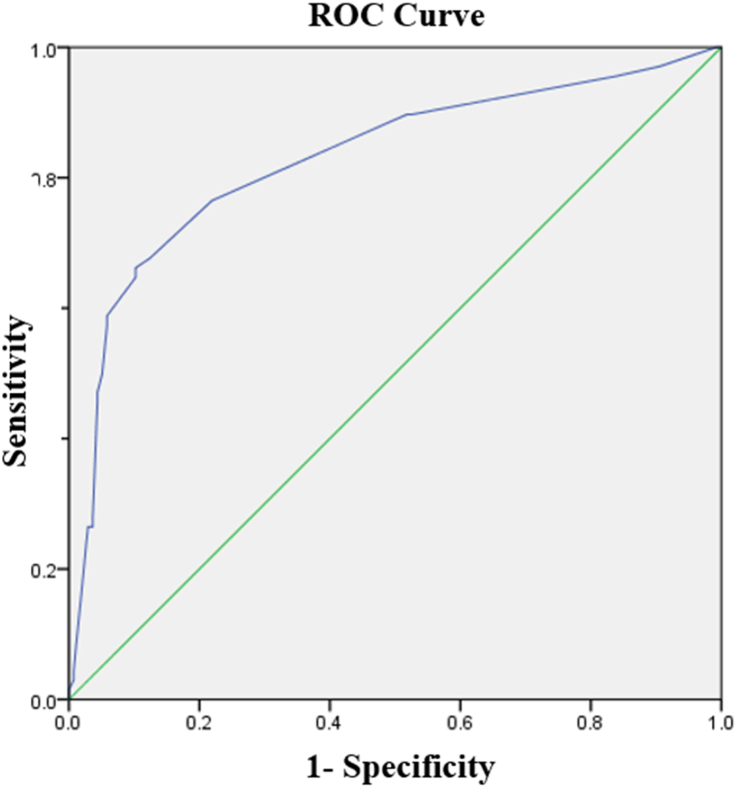
Receiver operating characteristic curve of postictal duration in predicting seizure recurrence within 6 h.

The performance of postictal duration in predicting seizure recurrence was tested using the ROC analysis (Fig. 2). The area under the curve was calculated as 0.829 (95% CI: 0.763–0.894). Using Youden's index, the cut-off value of postictal duration in predicting seizure recurrence with the highest sensitivity and specificity was found to be 22.5 min. At this cut-off value, a prolonged postictal state had a sensitivity of 66.2%, specificity of 89.8%, positive likelihood ratio of 6.5, negative likelihood ratio of 0.4, positive predictive value of 76.3%, negative predictive value of 84.3%, and accuracy of 82% in the prediction of seizure recurrence within 6 h.

Patients with generalized tonic-clonic seizures, those that had consumed alcohol within the last 24 h, those with a history of ischemic stroke, coronary artery disease and Alzheimer's disease as a comorbid disease, those with major neurological deficits as examination findings had a higher rate of seizure recurrence in the emergency department. Furthermore, it was observed that the patients with high glucose, C-reactive protein, phosphorus, lactate, and blood urea nitrogen levels, those with low potassium values, and those with an increased anion gap and osmolarity had higher seizure recurrence. However, the above-mentioned factors did not result in statistically significant differences.

## 6 Discussion

In this study, we investigated possible risk factors for epileptic seizure recurrence in patients presenting to the emergency department with epileptic seizures. We found postictal duration to be the only independent predictor of seizure recurrence in this patient population. Using Youden's index, the cut-off value of postictal duration in predicting seizure recurrence with the highest sensitivity and specificity was calculated as 22.5 min.

While epileptic seizures generally do not last very long, the postictal state may continue for up to several days [11]. Seizure recurrence may be affected by various factors, such as the prolongation of the postictal phase, disruption of the synchronization of neuronal networks, depletion of the energy substrate of neurons, and desensitization to inhibitory receptors may be mechanisms [11]. In a study by Ohira et al., it was found that patients older than 65 years of age, those with a low modified Rankin score, those with a prolonged seizure state, and those that did not regularly use their antiepileptic drugs had a longer postictal phase [12]. The number of patients with a prolonged postictal state is not small, and these patients may commonly present with behavioral impairment, confusion, and amnesia [13]. In eight cases reported by Fagan et al. and 10 cases reported by Bauer et al., the postictal state lasting up to 36 h was examined using electroencephalography (EEG), and sustained and recurrent ictal bursts were observed. This condition was associated with recurrent seizures and non-convulsive status epilepticus [14,15]. It can be very difficult to evaluate postictal duration in patients with epileptic seizures that have fully regained consciousness by the time they arrive at the emergency department. Patients or their relatives may not be able to state an exact duration. Therefore, we used the estimated duration stated by the relatives of patients who had had this type of seizure but regained consciousness when they were admitted to the emergency department. When the literature is reviewed, there are no data showing that a prolonged postictal state increases the risk of seizure recurrence.

A low GCS score at the time of presentation to the emergency department may be a predictor of seizure recurrence in the emergency department. Similar to our study, Sinha et al., evaluating patients with new-onset seizures in a population aged over 60 years, found that a low GCS score was a strong predictor of seizure cluster formation [16]. A lower GCS score might suggest more severe underlying illness or prolonged postictal duration and prolonged seizures. In another study, Choquet et al. defined seizures that recurred within 24 h as a seizure cluster and found that patients over the age of 40 years experienced seizure recurrence more frequently, as in our study. In contrast, in the Fisher study, no relationship was found between age and seizure cluster [17]. However, the large number of pediatric patents in that study may have affected the results. Increased risk of seizures in advanced age and drug incompatibility may also be associated with altered pharmacodynamic and pharmacokinetic effects of drugs, decreasing neurotransmitter activity.

The best parameter predicting seizure recurrence in the early period is EEG together with etiological factors [18]. It has also been suggested that the clustering of seizures may be an indicator of poorly controlled epilepsy [3]. Some patients with epilepsy have a higher risk of developing seizure clusters than others. A history of seizure clusters or status epilepticus, head trauma, earlier age of seizure onset, and a history of high seizure frequency (one or more seizures per week) within the first 12 months after the onset of epilepsy are other causes that have been associated with seizure clusters [5,[19], [20], [21], [22], [23], [24], [25]]. The most important risk factor for seizure clusters is having persistent epilepsy with a high mean seizure frequency [26]. Other triggering factors have been reported as sleep deprivation, stress, febrile disease, missing or changing medication, alcohol consumption, and menstruation [22]. Discrepancies in reported results may be related to the differences in study methodologies. However, seizure clusters can occur even in the absence of any trigger [26].

Kılıç et al. suggested that there was no laboratory parameter that could predict whether patients presenting with epileptic seizures would have recurrent episodes [27]. Furthermore, Bozan et al., evaluating 102 patients, found that no laboratory parameter was able to predict seizure recurrence in patients presenting to the emergency department with the complaint of epileptic seizures [28]. Similarly, in our study, although we observed that patients with high glucose, C-reactive protein, phosphorus, blood urea nitrogen, and lactate values, increased anion gap and high osmolarity, and low potassium levels had recurrent seizures at a higher rate, but no laboratory parameter significantly predicted seizures in the multivariable analysis.

The patients who should undergo antiepileptic drug loading to prevent recurrent seizures in the emergency department and the effectiveness of these drugs have not yet been examined in controlled studies [29]. This situation further complicates the process of determining seizure control strategies in the emergency department. Factors such as advanced age, low Glasgow Coma Scale (GCS) score at admission, and prolonged postictal duration should be considered when contemplating antiepileptic treatment. The lack of data on antiepileptic drug loading for preventing recurrent seizures in the emergency department leads to significant gaps in clinical evidence, creating an important need for future research and guidance in clinical applications.

## 7 Limitations

One of the most important limitations of this study is that it was carried out in a single center. Furthermore, at the time of the study, the emergency department admission profiles of the patients were different due to the COVID-19 pandemic. Additionally, the inability to measure serum alcohol and drug levels in the clinic where the study was conducted is another limitation. Lastly, we were not able to evaluate the antiepileptic drug levels of the patients.

## 8 Conclusions

In conclusion, we observed that a prolonged postictal duration, low GCS score, and advanced age were the independent predictors of seizure recurrence in patients presenting to the emergency department with the complaint of epileptic seizures. We consider that patients with a prolonged postictal state, low GCS score, and advanced age should be more carefully followed up for seizure recurrence.

## 9 Article summary

1. Why is this topic important?

The number of patients who have had epileptic seizures and applied to the emergency department is not small, but the information about the emergency department management of the patients who applied to the emergency department with the complaint of epileptic seizures is limited.

2. What does this study attempt to show?

In this study, it was aimed to investigate the factors affecting the recurrence of epileptic seizures in the follow-up of patients presenting to the emergency department with the complaint of epileptic seizures.

3. What are the key findings?

Prolonged postictal duration, low GCS score, and advanced age were the independent predictors of seizure recurrence in patients presenting to the emergency department with the complaint of epileptic seizures. The cut-off value of postictal duration in predicting seizure recurrence at the highest sensitivity (66.2%) and specificity (89.8%) was 22.5 min.

4. How is patient care impacted?

We speculate that the patients with a prolonged postictal state, low GCS score, and advanced age should be more carefully followed up for seizure recurrence.

## CRediT authorship contribution statement

**Umit Can Dolek:** Writing – review & editing, Writing – original draft, Visualization, Supervision, Software, Resources, Project administration, Methodology, Investigation, Funding acquisition, Formal analysis, Data curation, Conceptualization. **Mustafa Gokce:** Writing – review & editing, Writing – original draft, Conceptualization. **Mehmet Muzaffer Islam:** Software, Formal analysis. **Serdar Ozdemir:** Writing – review & editing, Writing – original draft. **Gokhan Aksel:** Visualization, Supervision, Software, Methodology. **Abdullah Algin:** Methodology, Formal analysis.

## Declaration of competing interest

The authors declare that they have no known competing financial interests or personal relationships that could have appeared to influence the work reported in this paper.
